# Supplementary material for: The complete mitogenome of Arion vulgaris Moquin-Tandon, 1855 (Gastropoda: Stylommatophora): mitochondrial genome architecture, evolution and phylogenetic considerations within Stylommatophora
Source: PeerJ. 2020 Feb 21;8:e8603. doi: 10.7717/peerj.8603 (PMC7039129; doi:10.7717/peerj.8603)
Supplement: Table S1 [file peerj-08-8603-s001.docx]

**Table S1.** Summary of best fit partition schemes and nucleotide substitution models

|  | **Partition scheme** | **Model** |
| --- | --- | --- |
| **P1** | *ATP6* 1st + *ND1* 1st + *ND2* 1st + *ND3* 1st + *ND4* 1st + *ND4L* 1st + *ND5* 1st + *ND6* 1st | GTR + I + G |
| **P2** | *ATP6* 2nd + *CYTB* 2nd + *ND1* 2nd + *ND2* 2nd + *ND3* 2nd + *ND4* 2nd + *ND4L* 2nd + *ND5* 2nd + *ND6* 2nd | GTR + I + G |
| **P3** | *ATP6* 3rd + *COX1* 3rd + *COX2* 3rd + *COX3* 3rd + *CYTB* 3rd + *ND1* 3rd + *ND2* 3rd + *ND3* 3rd + *ND4* 3rd + *ND4L* 3rd + *ND5* 3rd + *ND6* 3rd | HKY + I + G |
| **P4** | *ATP8* 1st + *ATP8* 2nd + *ATP8* 3rd + *rrnL* + *rrnS* + *trnA* + *trnR* + *trnN* + *trnD* + *trnC* + *trnQ* + *trnE* + *trnG* + *trnH* + *trnI* + *trnL1* + *trnL2* + *trnK* + *trnM* + *trnF* + *trnP* + *trnS1* + *trnS2* + *trnT* + *trnW* + *trnY* + *trnV* | GTR + I + G |
| **P5** | *COX1* 1st + *COX2* 1st + *COX3* 1st + *CYTB* 1st | GTR + I + G |
| **P6** | *COX1* 2nd + *COX2* 2nd + *COX3* 2nd | GTR + I + G |
