# Supplementary material for: The complete mitogenome of Arion vulgaris Moquin-Tandon, 1855 (Gastropoda: Stylommatophora): mitochondrial genome architecture, evolution and phylogenetic considerations within Stylommatophora
Source: PeerJ. 2020 Feb 21;8:e8603. doi: 10.7717/peerj.8603 (PMC7039129; doi:10.7717/peerj.8603)
Supplement: Table S2 [file peerj-08-8603-s002.docx]

**Table S2.** Regression of the pairwise distances of the first and second codon positions of each PCG, all positions of RNA genes and the first, second, first and second, and third codon positions of the concatenated mitochondrial PCGs.

| **Gene** | **Regression** | **R** | **Average GTR distance** |
| --- | --- | --- | --- |
| *ATP6* | y = 0.0626x + 0.274 | 0.7819 | 1.5624 |
| *ATP8* | y = 0.0001x + 0.3919 | 0.5420 | 1067.7146 |
| *COX1* | y = 0.6084x + 0.0221 | 0.9935 | 0.1611 |
| *COX2* | y = 0.1909x + 0.1527 | 0.8960 | 0.5734 |
| *COX3* | y = 0.3148x + 0.0879 | 0.9620 | 0.4283 |
| *CYTB* | y = 0.3148x + 0.0921 | 0.9594 | 0.4709 |
| *ND1* | y = 0.1695x + 0.1643 | 0.9133 | 0.9260 |
| *ND2* | y = 9E-06x + 0.4495 | 0.4323 | 2040.3701 |
| *ND3* | y = 2E-05x + 0.3981 | 0.5514 | 1127.3637 |
| *ND4* | y = 0.0642x + 0.2721 | 0.7938 | 1.7590 |
| *ND4L* | y = 2E-05x + 0.4225 | 0.5594 | 4150.3960 |
| *ND5* | y = 0.0465x + 0.3076 | 0.7014 | 1.7954 |
| *ND6* | y = 0.0002x + 0.3434 | 0.6349 | 937.2557 |
| rRNAs | y = 0.0499x + 0.2856 | 0.7631 | 2.0819 |
| tRNAs | y = 0.0236x + 0.3405 | 0.6491 | 2.5675 |
| 1st codon position | y = 0.0712x + 0.2618 | 0.8148 | 1.7962 |
| 2nd codon position | y = 0.2507x + 0.1118 | 0.9456 | 0.5990 |
| 1st and 2nd codon position | y = 0.1812x + 0.1606 | 0.9168 | 0.9114 |
| 3rd codon position | y = 0.0272x + 0.0951 | 0.4202 | 17.2012 |
