# Supplementary material for: The complete mitogenome of Arion vulgaris Moquin-Tandon, 1855 (Gastropoda: Stylommatophora): mitochondrial genome architecture, evolution and phylogenetic considerations within Stylommatophora
Source: PeerJ. 2020 Feb 21;8:e8603. doi: 10.7717/peerj.8603 (PMC7039129; doi:10.7717/peerj.8603)
Supplement: Table S3 [file peerj-08-8603-s003.docx]

**Table S3.** Nucleotide compositions and skewness values of whole mitogenomes of stylommatophoran species

| **Species** | **T%** | **C%** | **A%** | **G%** | **A+T%** | **AT-skew** | **GC-skew** |
| --- | --- | --- | --- | --- | --- | --- | --- |
| *Achatina fulica* | 35.47 | 17.10 | 27.97 | 19.46 | 63.44 | -0.1183 | 0.0645 |
| *Achatinella fulgens* | 42.55 | 9.27 | 36.38 | 11.79 | 78.94 | -0.0782 | 0.1194 |
| *Achatinella mustelina* | 42.76 | 8.83 | 37.31 | 11.10 | 80.07 | -0.0680 | 0.1140 |
| *Achatinella sowerbyana* | 42.61 | 9.18 | 36.51 | 11.70 | 79.12 | -0.0770 | 0.1202 |
| *Aegista aubryana* | 37.86 | 14.45 | 31.32 | 16.36 | 69.18 | -0.0946 | 0.0620 |
| *Aegista diversifamilia* | 38.59 | 13.26 | 32.48 | 15.66 | 71.07 | -0.0860 | 0.0830 |
| *Albinaria caerulea* | 37.90 | 13.81 | 32.75 | 15.54 | 70.65 | -0.0728 | 0.0591 |
| *Arion rufus* | 37.14 | 14.61 | 32.16 | 16.09 | 69.30 | -0.0720 | 0.0485 |
| *Arion vulgaris* | 37.75 | 14.26 | 32.45 | 15.54 | 70.20 | -0.0756 | 0.0431 |
| *Camaena cicatricosa* | 37.90 | 13.47 | 31.90 | 16.72 | 69.80 | -0.0860 | 0.1077 |
| *Camaena poyuensis* | 38.09 | 13.31 | 31.29 | 17.31 | 69.38 | -0.0980 | 0.1304 |
| *Cepaea nemoralis* | 33.63 | 18.94 | 26.16 | 21.26 | 59.79 | -0.1249 | 0.0577 |
| *Cerion incanum* | 35.97 | 15.83 | 29.75 | 18.45 | 65.72 | -0.0948 | 0.0765 |
| *Cerion tridentatum costellata* | 36.08 | 15.62 | 28.18 | 20.11 | 64.27 | -0.1229 | 0.1257 |
| *Cerion uva* | 34.45 | 17.29 | 28.29 | 19.98 | 62.73 | -0.0982 | 0.0721 |
| *Cernuella virgata* | 36.89 | 15.59 | 29.07 | 18.46 | 65.96 | -0.1186 | 0.0843 |
| *Cornu aspersum* | 39.15 | 13.61 | 30.72 | 16.52 | 69.87 | -0.1207 | 0.0966 |
| *Cylindrus obtusus* | 35.76 | 16.61 | 25.78 | 21.86 | 61.53 | -0.1622 | 0.1367 |
| *Deroceras reticulatum* | 39.14 | 12.17 | 31.04 | 17.65 | 70.17 | -0.1154 | 0.1838 |
| *Dolicheulota formosensis* | 41.81 | 13.12 | 28.38 | 16.70 | 70.18 | -0.1914 | 0.1199 |
| *Gastrocopta cristata* | 38.39 | 13.62 | 30.80 | 17.19 | 69.19 | -0.1096 | 0.1159 |
| *Helicella itala* | 37.28 | 15.27 | 28.94 | 18.51 | 66.22 | -0.1260 | 0.0958 |
| *Helix pomatia* | 37.41 | 15.07 | 29.60 | 17.92 | 67.01 | -0.1166 | 0.0862 |
| *Mastigeulota kiangsinensis* | 37.91 | 14.38 | 29.48 | 18.22 | 67.40 | -0.1251 | 0.1176 |
| *Meghimatium bilineatum* | 39.55 | 13.92 | 31.89 | 14.64 | 71.44 | -0.1072 | 0.0251 |
| *Microceramus pontificus* | 39.06 | 12.29 | 32.88 | 15.77 | 71.94 | -0.0860 | 0.1238 |
| *Naesiotus nux* | 39.69 | 12.03 | 33.57 | 14.71 | 73.26 | -0.0834 | 0.1004 |
| *Orcula dolium* | 35.77 | 16.28 | 30.21 | 17.74 | 65.98 | -0.0844 | 0.0431 |
| *Partulina redfieldi* | 42.44 | 8.87 | 37.23 | 11.46 | 79.67 | -0.0654 | 0.1274 |
| *Philomycus bilineatus* | 39.44 | 13.69 | 32.68 | 14.20 | 72.11 | -0.0938 | 0.0182 |
| *Polygyra cereolus* | 39.78 | 12.58 | 28.85 | 18.78 | 68.64 | -0.1592 | 0.1978 |
| *Praticolella mexicana* | 39.42 | 13.01 | 28.57 | 19.00 | 67.99 | -0.1596 | 0.1869 |
| *Pupilla muscorum* | 39.27 | 12.93 | 32.52 | 15.27 | 71.79 | -0.0941 | 0.0829 |
| *Succinea putris* | 43.06 | 10.87 | 33.94 | 12.13 | 77.00 | -0.1185 | 0.0551 |
| *Vertigo pusilla* | 39.66 | 12.27 | 32.57 | 15.49 | 72.23 | -0.0982 | 0.1159 |
