# Supplementary material for: The complete mitogenome of Arion vulgaris Moquin-Tandon, 1855 (Gastropoda: Stylommatophora): mitochondrial genome architecture, evolution and phylogenetic considerations within Stylommatophora
Source: PeerJ. 2020 Feb 21;8:e8603. doi: 10.7717/peerj.8603 (PMC7039129; doi:10.7717/peerj.8603)
Supplement: Table S4 [file peerj-08-8603-s004.docx]

**Table S4.** Nucleotide and amino acid identities of mitochondrial protein coding genes among stylommatophoran and between *Arion* species

|  | **Among Stylommatophora** | |  | **Between *Arion* species** | |
| --- | --- | --- | --- | --- | --- |
| **Genes** | **Nucleotide identity (%)** | **Amino acid identity (%)** |  | **Nucleotide identity (%)** | **Amino acid identity (%)** |
| *ATP6* | 20.39 | 22.36 |  | 79.39 | 81.36 |
| *ATP8* | 34.73 | 28.57 |  | 74.24 | 68.18 |
| *COX1* | 41.94 | 56.45 |  | 87.56 | 97.45 |
| *COX2* | 32.30 | 35.14 |  | 86.94 | 96.40 |
| *COX3* | 36.69 | 42.38 |  | 86.92 | 94.23 |
| *CYTB* | 29.14 | 34.76 |  | 84.87 | 89.42 |
| *ND1* | 22.78 | 25.15 |  | 84.78 | 90.00 |
| *ND2* | 19.18 | 17.71 |  | 79.14 | 78.93 |
| *ND3* | 24.04 | 21.09 |  | 78.84 | 80.00 |
| *ND4* | 16.87 | 16.67 |  | 83.30 | 85.81 |
| *ND4L* | 28.72 | 25.16 |  | 84.00 | 83.00 |
| *ND5* | 18.02 | 19.83 |  | 82.33 | 84.52 |
| *ND6* | 15.20 | 11.92 |  | 83.23 | 79.62 |
