# Supplementary material for: The complete mitogenome of Arion vulgaris Moquin-Tandon, 1855 (Gastropoda: Stylommatophora): mitochondrial genome architecture, evolution and phylogenetic considerations within Stylommatophora
Source: PeerJ. 2020 Feb 21;8:e8603. doi: 10.7717/peerj.8603 (PMC7039129; doi:10.7717/peerj.8603)
Supplement: Table S5 [file peerj-08-8603-s005.docx]

**Table S5.** Codon usages of the mitochondrial PCGs of stylommatophoran species.

|  | *A. vulgaris* | | *A. rufus* | | *A. fulica* | | *A. fulgens* | | *A. mustelina* | | *A. sowerbyana* | | *A. aubryana* | | *A. diversifamilia* | | *A. caerulea* | |
| --- | --- | --- | --- | --- | --- | --- | --- | --- | --- | --- | --- | --- | --- | --- | --- | --- | --- | --- |
| Codon | Count | RSCU | Count | RSCU | Count | RSCU | Count | RSCU | Count | RSCU | Count | RSCU | Count | RSCU | Count | RSCU | Count | RSCU |
| UUU(F) | 226 | 1.64 | 215 | 1.55 | 218 | 1.48 | 345 | 1.93 | 358 | 1.95 | 354 | 1.90 | 263 | 1.67 | 269 | 1.74 | 241 | 1.81 |
| UUC(F) | 50 | 0.36 | 63 | 0.45 | 75 | 0.52 | 12 | 0.07 | 9 | 0.05 | 18 | 0.10 | 52 | 0.33 | 40 | 0.26 | 25 | 0.19 |
| UUA(L) | 389 | 3.89 | 347 | 3.64 | 215 | 2.31 | 456 | 4.59 | 472 | 4.77 | 465 | 4.77 | 321 | 3.23 | 363 | 3.70 | 341 | 3.38 |
| UUG(L) | 36 | 0.36 | 54 | 0.57 | 73 | 0.79 | 29 | 0.28 | 23 | 0.23 | 33 | 0.33 | 75 | 0.75 | 42 | 0.43 | 62 | 0.63 |
| CUU(L) | 85 | 0.85 | 71 | 0.74 | 96 | 1.04 | 77 | 0.78 | 74 | 0.75 | 68 | 0.69 | 90 | 0.90 | 96 | 0.98 | 83 | 0.83 |
| CUC(L) | 8 | 0.08 | 14 | 0.15 | 35 | 0.38 | 5 | 0.05 | 1 | 0.01 | 3 | 0.02 | 18 | 0.18 | 10 | 0.11 | 14 | 0.14 |
| CUA(L) | 71 | 0.71 | 73 | 0.77 | 104 | 1.12 | 27 | 0.27 | 18 | 0.18 | 16 | 0.16 | 74 | 0.74 | 68 | 0.69 | 79 | 0.79 |
| CUG(L) | 11 | 0.11 | 13 | 0.14 | 34 | 0.37 | 3 | 0.03 | 7 | 0.06 | 3 | 0.03 | 20 | 0.19 | 8 | 0.08 | 23 | 0.24 |
| AUU(I) | 230 | 1.74 | 218 | 1.71 | 192 | 1.49 | 335 | 1.94 | 326 | 1.96 | 309 | 1.91 | 229 | 1.79 | 231 | 1.82 | 267 | 1.76 |
| AUC(I) | 35 | 0.26 | 37 | 0.29 | 66 | 0.51 | 10 | 0.06 | 7 | 0.04 | 16 | 0.09 | 27 | 0.21 | 23 | 0.18 | 36 | 0.24 |
| AUA(M) | 213 | 1.66 | 213 | 1.61 | 157 | 1.44 | 285 | 1.83 | 282 | 1.87 | 289 | 1.83 | 215 | 1.75 | 237 | 1.79 | 213 | 1.62 |
| AUG(M) | 43 | 0.34 | 51 | 0.39 | 61 | 0.56 | 26 | 0.17 | 20 | 0.13 | 27 | 0.17 | 31 | 0.25 | 29 | 0.21 | 49 | 0.38 |
| GUU(V) | 71 | 1.17 | 79 | 1.24 | 124 | 1.50 | 74 | 1.48 | 81 | 1.64 | 86 | 1.63 | 105 | 1.48 | 112 | 1.64 | 90 | 1.57 |
| GUC(V) | 16 | 0.26 | 11 | 0.17 | 51 | 0.62 | 10 | 0.22 | 4 | 0.08 | 7 | 0.11 | 22 | 0.31 | 7 | 0.09 | 20 | 0.35 |
| GUA(V) | 110 | 1.82 | 117 | 1.84 | 99 | 1.20 | 108 | 2.16 | 105 | 2.15 | 107 | 2.03 | 116 | 1.63 | 124 | 1.80 | 98 | 1.69 |
| GUG(V) | 45 | 0.74 | 47 | 0.74 | 57 | 0.69 | 7 | 0.14 | 7 | 0.12 | 12 | 0.23 | 42 | 0.58 | 33 | 0.48 | 22 | 0.38 |
| UCU(S) | 99 | 2.13 | 84 | 1.84 | 86 | 1.69 | 108 | 2.38 | 96 | 1.97 | 92 | 1.95 | 55 | 1.33 | 73 | 1.71 | 77 | 1.79 |
| UCC(S) | 30 | 0.65 | 32 | 0.70 | 39 | 0.76 | 10 | 0.22 | 13 | 0.27 | 7 | 0.15 | 20 | 0.48 | 14 | 0.33 | 16 | 0.35 |
| UCA(S) | 52 | 1.12 | 58 | 1.27 | 98 | 1.90 | 64 | 1.41 | 81 | 1.67 | 83 | 1.76 | 59 | 1.40 | 65 | 1.52 | 68 | 1.58 |
| UCG(S) | 9 | 0.19 | 10 | 0.22 | 20 | 0.39 | 7 | 0.13 | 4 | 0.08 | 1 | 0.02 | 18 | 0.43 | 9 | 0.21 | 14 | 0.32 |
| CCU(P) | 54 | 1.16 | 46 | 1.01 | 55 | 1.55 | 68 | 2.31 | 70 | 2.37 | 72 | 2.44 | 66 | 1.87 | 66 | 1.80 | 49 | 1.48 |
| CCC(P) | 15 | 0.32 | 18 | 0.39 | 16 | 0.45 | 3 | 0.10 | 1 | 0.03 | 1 | 0.03 | 17 | 0.48 | 10 | 0.27 | 22 | 0.67 |
| CCA(P) | 72 | 1.55 | 77 | 1.68 | 53 | 1.49 | 43 | 1.46 | 44 | 1.49 | 43 | 1.46 | 51 | 1.45 | 60 | 1.63 | 49 | 1.52 |
| CCG(P) | 40 | 0.86 | 41 | 0.90 | 18 | 0.51 | 4 | 0.14 | 3 | 0.10 | 3 | 0.07 | 7 | 0.20 | 10 | 0.30 | 10 | 0.33 |
| ACU(T) | 80 | 1.71 | 70 | 1.43 | 62 | 1.41 | 73 | 1.97 | 74 | 1.90 | 73 | 1.96 | 87 | 1.72 | 94 | 1.98 | 77 | 1.66 |
| ACC(T) | 15 | 0.32 | 30 | 0.61 | 39 | 0.89 | 3 | 0.08 | 4 | 0.10 | 7 | 0.16 | 29 | 0.55 | 16 | 0.32 | 23 | 0.50 |
| ACA(T) | 82 | 1.75 | 83 | 1.69 | 60 | 1.36 | 66 | 1.78 | 73 | 1.87 | 68 | 1.80 | 61 | 1.21 | 70 | 1.49 | 73 | 1.58 |
| ACG(T) | 10 | 0.21 | 13 | 0.27 | 16 | 0.34 | 7 | 0.16 | 5 | 0.13 | 3 | 0.08 | 26 | 0.51 | 10 | 0.21 | 12 | 0.26 |
| GCU(A) | 88 | 1.91 | 83 | 1.83 | 85 | 1.94 | 73 | 2.21 | 74 | 2.21 | 72 | 2.17 | 85 | 1.73 | 78 | 1.72 | 91 | 1.89 |
| GCC(A) | 27 | 0.59 | 35 | 0.77 | 29 | 0.67 | 3 | 0.09 | 4 | 0.12 | 7 | 0.18 | 21 | 0.43 | 14 | 0.31 | 23 | 0.50 |
| GCA(A) | 55 | 1.20 | 44 | 0.97 | 43 | 0.99 | 51 | 1.55 | 52 | 1.55 | 49 | 1.50 | 64 | 1.32 | 72 | 1.59 | 66 | 1.37 |
| GCG(A) | 1 | 0.04 | 4 | 0.15 | 17 | 0.39 | 5 | 0.15 | 4 | 0.12 | 5 | 0.15 | 25 | 0.52 | 17 | 0.38 | 12 | 0.25 |
| UAU(Y) | 126 | 1.40 | 129 | 1.35 | 79 | 1.05 | 176 | 1.85 | 191 | 1.89 | 174 | 1.82 | 120 | 1.53 | 142 | 1.72 | 148 | 1.59 |
| UAC(Y) | 54 | 0.60 | 62 | 0.65 | 72 | 0.95 | 14 | 0.15 | 10 | 0.11 | 17 | 0.18 | 36 | 0.47 | 23 | 0.28 | 38 | 0.41 |
| CAU(H) | 42 | 1.15 | 62 | 1.59 | 42 | 1.14 | 60 | 1.82 | 62 | 1.85 | 64 | 1.88 | 57 | 1.58 | 61 | 1.69 | 61 | 1.65 |
| CAC(H) | 31 | 0.85 | 16 | 0.41 | 31 | 0.86 | 7 | 0.18 | 5 | 0.15 | 4 | 0.12 | 16 | 0.42 | 10 | 0.31 | 13 | 0.35 |
| CAA(Q) | 53 | 1.66 | 50 | 1.82 | 21 | 0.91 | 48 | 1.88 | 43 | 1.87 | 47 | 1.84 | 43 | 1.72 | 42 | 1.58 | 42 | 1.61 |
| CAG(Q) | 11 | 0.34 | 5 | 0.18 | 25 | 1.09 | 3 | 0.12 | 3 | 0.13 | 4 | 0.16 | 7 | 0.28 | 10 | 0.42 | 10 | 0.39 |
| AAU(N) | 106 | 1.64 | 113 | 1.61 | 56 | 1.29 | 146 | 1.87 | 150 | 1.84 | 142 | 1.80 | 98 | 1.50 | 118 | 1.71 | 107 | 1.54 |
| AAC(N) | 23 | 0.36 | 27 | 0.39 | 31 | 0.71 | 10 | 0.13 | 13 | 0.16 | 16 | 0.20 | 33 | 0.50 | 20 | 0.29 | 33 | 0.46 |
| AAA(K) | 62 | 1.51 | 62 | 1.53 | 48 | 1.25 | 109 | 1.74 | 118 | 1.82 | 105 | 1.75 | 77 | 1.67 | 81 | 1.65 | 73 | 1.70 |
| AAG(K) | 20 | 0.49 | 19 | 0.47 | 29 | 0.75 | 16 | 0.26 | 12 | 0.18 | 16 | 0.25 | 16 | 0.33 | 17 | 0.35 | 13 | 0.30 |
| GAU(D) | 49 | 1.66 | 45 | 1.58 | 46 | 1.44 | 49 | 1.75 | 48 | 2.00 | 52 | 1.89 | 47 | 1.57 | 55 | 1.74 | 47 | 1.52 |
| GAC(D) | 10 | 0.34 | 12 | 0.42 | 18 | 0.56 | 7 | 0.25 | 0 | 0.00 | 3 | 0.11 | 13 | 0.43 | 8 | 0.26 | 16 | 0.48 |
| GAA(E) | 56 | 1.42 | 45 | 1.18 | 33 | 0.85 | 66 | 1.69 | 72 | 1.92 | 65 | 1.78 | 48 | 1.37 | 43 | 1.19 | 48 | 1.32 |
| GAG(E) | 23 | 0.58 | 31 | 0.82 | 43 | 1.15 | 12 | 0.31 | 3 | 0.08 | 8 | 0.22 | 22 | 0.63 | 29 | 0.81 | 25 | 0.68 |
| UGU(C) | 31 | 1.55 | 40 | 1.70 | 46 | 1.30 | 39 | 1.90 | 42 | 1.91 | 39 | 1.77 | 36 | 1.57 | 39 | 1.59 | 34 | 1.55 |
| UGC(C) | 9 | 0.45 | 7 | 0.30 | 23 | 0.70 | 3 | 0.10 | 3 | 0.09 | 5 | 0.23 | 10 | 0.43 | 10 | 0.41 | 10 | 0.45 |
| UGA(W) | 69 | 1.48 | 72 | 1.50 | 48 | 1.07 | 77 | 1.88 | 75 | 1.79 | 72 | 1.71 | 60 | 1.36 | 70 | 1.57 | 68 | 1.55 |
| UGG(W) | 24 | 0.52 | 24 | 0.50 | 42 | 0.93 | 5 | 0.12 | 9 | 0.21 | 12 | 0.29 | 29 | 0.64 | 20 | 0.43 | 20 | 0.45 |
| CGU(R) | 25 | 1.82 | 28 | 1.81 | 16 | 1.07 | 14 | 1.17 | 16 | 1.25 | 12 | 0.96 | 26 | 1.58 | 20 | 1.27 | 17 | 1.39 |
| CGC(R) | 10 | 0.73 | 7 | 0.45 | 8 | 0.57 | 0 | 0.00 | 0 | 0.00 | 3 | 0.24 | 9 | 0.55 | 4 | 0.25 | 4 | 0.33 |
| CGA(R) | 17 | 1.24 | 21 | 1.35 | 18 | 1.29 | 33 | 2.67 | 29 | 2.42 | 34 | 2.72 | 20 | 1.21 | 25 | 1.59 | 22 | 1.80 |
| CGG(R) | 3 | 0.22 | 6 | 0.39 | 16 | 1.07 | 3 | 0.17 | 4 | 0.33 | 1 | 0.08 | 10 | 0.67 | 14 | 0.89 | 7 | 0.49 |
| AGU(S) | 71 | 2.09 | 58 | 1.76 | 33 | 0.65 | 52 | 1.15 | 69 | 1.42 | 55 | 1.15 | 75 | 1.81 | 73 | 1.71 | 49 | 1.14 |
| AGC(S) | 21 | 0.62 | 26 | 0.79 | 23 | 0.45 | 8 | 0.18 | 9 | 0.19 | 5 | 0.11 | 20 | 0.48 | 20 | 0.45 | 29 | 0.65 |
| AGA(S) | 34 | 1.00 | 31 | 0.94 | 51 | 1.00 | 101 | 2.25 | 109 | 2.24 | 114 | 2.44 | 72 | 1.71 | 64 | 1.50 | 64 | 1.48 |
| AGG(S) | 10 | 0.29 | 17 | 0.52 | 59 | 1.16 | 13 | 0.29 | 8 | 0.16 | 20 | 0.42 | 16 | 0.36 | 23 | 0.56 | 30 | 0.70 |
| GGU(G) | 71 | 1.30 | 76 | 1.41 | 74 | 1.17 | 81 | 1.58 | 85 | 1.71 | 74 | 1.51 | 90 | 1.48 | 75 | 1.23 | 73 | 1.33 |
| GGC(G) | 19 | 0.35 | 21 | 0.39 | 35 | 0.56 | 5 | 0.10 | 5 | 0.10 | 9 | 0.18 | 23 | 0.38 | 16 | 0.24 | 23 | 0.44 |
| GGA(G) | 70 | 1.28 | 76 | 1.41 | 48 | 0.76 | 99 | 1.95 | 101 | 2.05 | 95 | 1.94 | 81 | 1.31 | 98 | 1.57 | 79 | 1.44 |
| GGG(G) | 14 | 0.32 | 28 | 0.62 | 95 | 1.51 | 20 | 0.37 | 7 | 0.14 | 18 | 0.37 | 51 | 0.84 | 59 | 0.96 | 43 | 0.79 |

**Table S5.** (continued) Codon usages of the mitochondrial PCGs of stylommatophoran species.

|  | *C. cicatricosa* | | *C. poyuensis* | | *C. nemoralis* | | *C. incanum* | | *C. tridentatum* | | *C. uva* | | *C. virgata* | | *C. aspersum* | | *C. obtusus* | |
| --- | --- | --- | --- | --- | --- | --- | --- | --- | --- | --- | --- | --- | --- | --- | --- | --- | --- | --- |
| Codon | Count | RSCU | Count | RSCU | Count | RSCU | Count | RSCU | Count | RSCU | Count | RSCU | Count | RSCU | Count | RSCU | Count | RSCU |
| UUU(F) | 251 | 1.79 | 250 | 1.79 | 216 | 1.47 | 198 | 1.51 | 224 | 1.68 | 195 | 1.52 | 251 | 1.67 | 278 | 1.72 | 265 | 1.66 |
| UUC(F) | 30 | 0.21 | 30 | 0.21 | 77 | 0.53 | 64 | 0.49 | 42 | 0.32 | 61 | 0.48 | 49 | 0.33 | 46 | 0.28 | 55 | 0.34 |
| UUA(L) | 374 | 3.83 | 384 | 3.89 | 164 | 1.66 | 263 | 2.77 | 265 | 2.74 | 226 | 2.37 | 335 | 3.04 | 332 | 3.48 | 269 | 2.47 |
| UUG(L) | 72 | 0.73 | 74 | 0.75 | 103 | 1.05 | 72 | 0.76 | 99 | 1.02 | 87 | 0.91 | 82 | 0.75 | 61 | 0.64 | 86 | 0.79 |
| CUU(L) | 61 | 0.62 | 61 | 0.62 | 101 | 1.04 | 87 | 0.92 | 94 | 0.96 | 88 | 0.93 | 88 | 0.80 | 96 | 1.01 | 124 | 1.14 |
| CUC(L) | 5 | 0.05 | 8 | 0.08 | 55 | 0.56 | 29 | 0.29 | 30 | 0.31 | 30 | 0.31 | 30 | 0.27 | 22 | 0.23 | 35 | 0.32 |
| CUA(L) | 62 | 0.65 | 49 | 0.51 | 104 | 1.06 | 94 | 0.99 | 69 | 0.71 | 101 | 1.06 | 101 | 0.91 | 55 | 0.58 | 107 | 0.98 |
| CUG(L) | 12 | 0.12 | 16 | 0.15 | 62 | 0.64 | 26 | 0.27 | 23 | 0.25 | 40 | 0.42 | 26 | 0.24 | 5 | 0.05 | 33 | 0.30 |
| AUU(I) | 243 | 1.75 | 224 | 1.75 | 143 | 1.53 | 231 | 1.63 | 202 | 1.58 | 179 | 1.45 | 209 | 1.74 | 246 | 1.88 | 178 | 1.71 |
| AUC(I) | 34 | 0.25 | 33 | 0.25 | 44 | 0.47 | 52 | 0.37 | 53 | 0.42 | 69 | 0.55 | 31 | 0.26 | 16 | 0.12 | 30 | 0.29 |
| AUA(M) | 186 | 1.63 | 192 | 1.67 | 107 | 1.28 | 189 | 1.45 | 178 | 1.47 | 157 | 1.37 | 159 | 1.51 | 182 | 1.78 | 101 | 1.29 |
| AUG(M) | 42 | 0.37 | 38 | 0.33 | 60 | 0.72 | 72 | 0.55 | 65 | 0.53 | 73 | 0.63 | 52 | 0.49 | 23 | 0.22 | 56 | 0.71 |
| GUU(V) | 112 | 1.57 | 117 | 1.55 | 101 | 1.44 | 100 | 1.36 | 120 | 1.49 | 127 | 1.48 | 125 | 1.55 | 120 | 1.95 | 112 | 1.49 |
| GUC(V) | 13 | 0.18 | 20 | 0.27 | 42 | 0.58 | 26 | 0.35 | 33 | 0.41 | 46 | 0.52 | 33 | 0.40 | 20 | 0.33 | 31 | 0.41 |
| GUA(V) | 117 | 1.64 | 124 | 1.63 | 85 | 1.19 | 121 | 1.65 | 95 | 1.19 | 103 | 1.19 | 116 | 1.44 | 87 | 1.41 | 78 | 1.04 |
| GUG(V) | 44 | 0.62 | 42 | 0.54 | 56 | 0.79 | 47 | 0.64 | 72 | 0.90 | 70 | 0.81 | 49 | 0.61 | 20 | 0.31 | 79 | 1.05 |
| UCU(S) | 66 | 1.60 | 65 | 1.58 | 62 | 1.34 | 101 | 1.96 | 105 | 2.11 | 96 | 1.88 | 59 | 1.29 | 99 | 2.19 | 68 | 1.52 |
| UCC(S) | 10 | 0.24 | 13 | 0.32 | 42 | 0.87 | 38 | 0.73 | 33 | 0.64 | 29 | 0.55 | 21 | 0.46 | 18 | 0.40 | 33 | 0.74 |
| UCA(S) | 55 | 1.34 | 56 | 1.36 | 34 | 0.73 | 86 | 1.65 | 64 | 1.28 | 72 | 1.39 | 62 | 1.39 | 55 | 1.22 | 36 | 0.80 |
| UCG(S) | 14 | 0.34 | 13 | 0.32 | 30 | 0.64 | 14 | 0.27 | 16 | 0.32 | 20 | 0.37 | 14 | 0.31 | 8 | 0.18 | 18 | 0.40 |
| CCU(P) | 59 | 1.70 | 64 | 1.83 | 56 | 1.53 | 68 | 1.88 | 60 | 1.59 | 70 | 1.75 | 73 | 1.91 | 75 | 2.08 | 59 | 1.51 |
| CCC(P) | 13 | 0.37 | 10 | 0.29 | 33 | 0.88 | 25 | 0.69 | 46 | 1.19 | 44 | 1.10 | 30 | 0.77 | 9 | 0.25 | 42 | 1.05 |
| CCA(P) | 48 | 1.38 | 49 | 1.43 | 33 | 0.90 | 46 | 1.24 | 34 | 0.90 | 38 | 0.95 | 33 | 0.86 | 46 | 1.28 | 31 | 0.79 |
| CCG(P) | 20 | 0.55 | 16 | 0.46 | 25 | 0.68 | 7 | 0.19 | 12 | 0.32 | 8 | 0.20 | 17 | 0.46 | 14 | 0.39 | 25 | 0.64 |
| ACU(T) | 86 | 1.63 | 77 | 1.49 | 64 | 1.42 | 82 | 1.84 | 95 | 2.17 | 62 | 1.51 | 78 | 1.57 | 88 | 1.70 | 60 | 1.19 |
| ACC(T) | 20 | 0.36 | 20 | 0.39 | 27 | 0.60 | 30 | 0.67 | 29 | 0.64 | 36 | 0.89 | 30 | 0.59 | 29 | 0.56 | 35 | 0.70 |
| ACA(T) | 79 | 1.50 | 81 | 1.55 | 59 | 1.29 | 49 | 1.12 | 38 | 0.87 | 49 | 1.20 | 70 | 1.42 | 70 | 1.34 | 60 | 1.19 |
| ACG(T) | 27 | 0.51 | 30 | 0.58 | 31 | 0.69 | 16 | 0.36 | 14 | 0.32 | 17 | 0.41 | 21 | 0.42 | 21 | 0.40 | 46 | 0.92 |
| GCU(A) | 96 | 2.13 | 87 | 1.90 | 96 | 1.44 | 98 | 2.05 | 94 | 1.80 | 78 | 1.51 | 90 | 1.81 | 90 | 2.07 | 82 | 1.43 |
| GCC(A) | 10 | 0.24 | 22 | 0.48 | 57 | 0.85 | 33 | 0.70 | 36 | 0.70 | 51 | 0.99 | 39 | 0.79 | 20 | 0.44 | 36 | 0.65 |
| GCA(A) | 51 | 1.13 | 49 | 1.07 | 72 | 1.06 | 47 | 0.99 | 51 | 0.99 | 57 | 1.10 | 51 | 1.03 | 49 | 1.15 | 55 | 0.96 |
| GCG(A) | 22 | 0.49 | 25 | 0.55 | 43 | 0.64 | 12 | 0.25 | 27 | 0.52 | 21 | 0.41 | 18 | 0.37 | 16 | 0.34 | 55 | 0.96 |
| UAU(Y) | 134 | 1.58 | 139 | 1.58 | 107 | 1.15 | 107 | 1.37 | 111 | 1.38 | 98 | 1.25 | 148 | 1.47 | 148 | 1.55 | 121 | 1.37 |
| UAC(Y) | 36 | 0.42 | 36 | 0.42 | 79 | 0.85 | 49 | 0.63 | 49 | 0.63 | 59 | 0.75 | 53 | 0.53 | 43 | 0.45 | 56 | 0.63 |
| CAU(H) | 61 | 1.39 | 68 | 1.53 | 51 | 1.17 | 55 | 1.38 | 55 | 1.36 | 36 | 0.97 | 49 | 1.33 | 61 | 1.58 | 44 | 1.14 |
| CAC(H) | 27 | 0.61 | 21 | 0.47 | 36 | 0.83 | 23 | 0.62 | 26 | 0.64 | 38 | 1.03 | 25 | 0.67 | 16 | 0.42 | 33 | 0.86 |
| CAA(Q) | 52 | 1.79 | 55 | 1.86 | 39 | 1.28 | 40 | 1.27 | 42 | 1.24 | 29 | 1.06 | 42 | 1.43 | 31 | 1.24 | 33 | 1.16 |
| CAG(Q) | 7 | 0.21 | 4 | 0.14 | 22 | 0.72 | 23 | 0.73 | 25 | 0.76 | 25 | 0.94 | 17 | 0.57 | 20 | 0.76 | 23 | 0.84 |
| AAU(N) | 109 | 1.58 | 111 | 1.59 | 49 | 1.16 | 66 | 1.47 | 68 | 1.48 | 55 | 1.09 | 75 | 1.27 | 99 | 1.56 | 57 | 1.27 |
| AAC(N) | 29 | 0.42 | 29 | 0.41 | 36 | 0.84 | 23 | 0.53 | 23 | 0.52 | 46 | 0.91 | 43 | 0.73 | 29 | 0.44 | 33 | 0.73 |
| AAA(K) | 65 | 1.67 | 70 | 1.69 | 35 | 1.01 | 57 | 1.37 | 53 | 1.25 | 42 | 1.04 | 49 | 1.25 | 55 | 1.50 | 35 | 1.03 |
| AAG(K) | 13 | 0.33 | 13 | 0.31 | 34 | 0.99 | 26 | 0.63 | 33 | 0.75 | 39 | 0.96 | 30 | 0.75 | 18 | 0.50 | 33 | 0.97 |
| GAU(D) | 48 | 1.60 | 51 | 1.67 | 29 | 0.97 | 33 | 1.14 | 46 | 1.32 | 39 | 1.15 | 46 | 1.42 | 53 | 1.63 | 36 | 1.16 |
| GAC(D) | 12 | 0.40 | 10 | 0.33 | 31 | 1.03 | 25 | 0.86 | 23 | 0.68 | 29 | 0.85 | 18 | 0.58 | 12 | 0.37 | 26 | 0.84 |
| GAA(E) | 48 | 1.35 | 43 | 1.25 | 30 | 1.09 | 48 | 1.12 | 33 | 0.84 | 35 | 0.95 | 55 | 1.33 | 49 | 1.53 | 34 | 1.01 |
| GAG(E) | 23 | 0.65 | 26 | 0.75 | 25 | 0.91 | 38 | 0.88 | 44 | 1.16 | 39 | 1.05 | 27 | 0.67 | 16 | 0.47 | 33 | 0.99 |
| UGU(C) | 33 | 1.49 | 40 | 1.63 | 46 | 1.14 | 33 | 1.31 | 29 | 1.41 | 21 | 1.08 | 38 | 1.49 | 52 | 1.46 | 36 | 1.47 |
| UGC(C) | 10 | 0.51 | 9 | 0.37 | 34 | 0.86 | 17 | 0.69 | 12 | 0.59 | 18 | 0.92 | 13 | 0.51 | 20 | 0.54 | 13 | 0.53 |
| UGA(W) | 57 | 1.34 | 57 | 1.30 | 57 | 1.25 | 64 | 1.33 | 62 | 1.33 | 55 | 1.09 | 49 | 1.16 | 73 | 1.62 | 47 | 1.15 |
| UGG(W) | 29 | 0.66 | 31 | 0.70 | 34 | 0.75 | 33 | 0.67 | 31 | 0.67 | 46 | 0.91 | 36 | 0.84 | 17 | 0.38 | 35 | 0.85 |
| CGU(R) | 22 | 1.33 | 20 | 1.18 | 31 | 1.32 | 16 | 1.14 | 17 | 1.15 | 16 | 1.03 | 26 | 1.55 | 33 | 2.00 | 30 | 1.69 |
| CGC(R) | 7 | 0.42 | 9 | 0.53 | 22 | 0.94 | 8 | 0.57 | 3 | 0.14 | 9 | 0.58 | 10 | 0.65 | 10 | 0.63 | 13 | 0.73 |
| CGA(R) | 22 | 1.33 | 18 | 1.06 | 18 | 0.77 | 22 | 1.57 | 18 | 1.22 | 22 | 1.42 | 14 | 0.84 | 17 | 1.06 | 12 | 0.68 |
| CGG(R) | 16 | 0.91 | 21 | 1.24 | 23 | 0.98 | 10 | 0.71 | 22 | 1.49 | 16 | 0.97 | 17 | 0.97 | 5 | 0.31 | 16 | 0.90 |
| AGU(S) | 78 | 1.90 | 82 | 1.99 | 69 | 1.47 | 33 | 0.63 | 38 | 0.76 | 33 | 0.63 | 77 | 1.70 | 85 | 1.88 | 78 | 1.74 |
| AGC(S) | 27 | 0.66 | 22 | 0.53 | 53 | 1.13 | 23 | 0.46 | 29 | 0.56 | 35 | 0.69 | 23 | 0.50 | 31 | 0.69 | 47 | 1.05 |
| AGA(S) | 46 | 1.09 | 36 | 0.90 | 49 | 1.05 | 72 | 1.38 | 59 | 1.18 | 68 | 1.31 | 53 | 1.17 | 43 | 0.95 | 27 | 0.60 |
| AGG(S) | 34 | 0.83 | 42 | 1.02 | 36 | 0.77 | 47 | 0.90 | 57 | 1.14 | 60 | 1.18 | 53 | 1.17 | 23 | 0.51 | 52 | 1.16 |
| GGU(G) | 94 | 1.67 | 88 | 1.59 | 74 | 1.38 | 60 | 1.07 | 55 | 0.97 | 49 | 0.90 | 101 | 1.60 | 107 | 2.06 | 81 | 1.29 |
| GGC(G) | 21 | 0.37 | 40 | 0.72 | 57 | 1.06 | 25 | 0.45 | 23 | 0.41 | 44 | 0.79 | 39 | 0.62 | 22 | 0.43 | 59 | 0.94 |
| GGA(G) | 47 | 0.84 | 42 | 0.74 | 40 | 0.74 | 87 | 1.55 | 62 | 1.11 | 36 | 0.65 | 43 | 0.69 | 59 | 1.13 | 21 | 0.34 |
| GGG(G) | 62 | 1.12 | 53 | 0.95 | 44 | 0.82 | 52 | 0.93 | 86 | 1.52 | 94 | 1.67 | 69 | 1.09 | 20 | 0.39 | 88 | 1.44 |

**Table S5.** (continued) Codon usages of the mitochondrial PCGs of stylommatophoran species.

|  | *D. reticulatum* | | *D. formosensis* | | *G. cristata* | | *H. itala* | | *H. pomatia* | | *M. kiangsinensis* | | *M. bilineatum* | | *M. pontificus* | | *N. nux* | |
| --- | --- | --- | --- | --- | --- | --- | --- | --- | --- | --- | --- | --- | --- | --- | --- | --- | --- | --- |
| Codon | Count | RSCU | Count | RSCU | Count | RSCU | Count | RSCU | Count | RSCU | Count | RSCU | Count | RSCU | Count | RSCU | Count | RSCU |
| UUU(F) | 289 | 1.82 | 291 | 1.82 | 302 | 1.80 | 235 | 1.68 | 263 | 1.71 | 264 | 1.77 | 261 | 1.81 | 295 | 1.88 | 288 | 1.73 |
| UUC(F) | 29 | 0.18 | 29 | 0.18 | 34 | 0.20 | 44 | 0.32 | 44 | 0.29 | 34 | 0.23 | 27 | 0.19 | 20 | 0.12 | 45 | 0.27 |
| UUA(L) | 393 | 4.07 | 335 | 3.33 | 341 | 3.32 | 309 | 2.95 | 308 | 3.00 | 326 | 3.25 | 352 | 3.48 | 324 | 3.51 | 386 | 3.80 |
| UUG(L) | 68 | 0.69 | 78 | 0.77 | 69 | 0.67 | 92 | 0.88 | 75 | 0.73 | 83 | 0.83 | 57 | 0.56 | 78 | 0.84 | 57 | 0.57 |
| CUU(L) | 64 | 0.66 | 125 | 1.24 | 122 | 1.19 | 85 | 0.81 | 90 | 0.88 | 88 | 0.88 | 118 | 1.16 | 83 | 0.90 | 85 | 0.85 |
| CUC(L) | 5 | 0.05 | 10 | 0.11 | 16 | 0.16 | 21 | 0.20 | 36 | 0.35 | 20 | 0.19 | 14 | 0.15 | 7 | 0.06 | 14 | 0.14 |
| CUA(L) | 42 | 0.42 | 48 | 0.48 | 60 | 0.58 | 95 | 0.91 | 87 | 0.85 | 72 | 0.71 | 60 | 0.59 | 51 | 0.55 | 55 | 0.54 |
| CUG(L) | 10 | 0.10 | 7 | 0.07 | 9 | 0.09 | 26 | 0.25 | 21 | 0.20 | 14 | 0.14 | 7 | 0.06 | 12 | 0.13 | 10 | 0.10 |
| AUU(I) | 274 | 1.88 | 215 | 1.85 | 255 | 1.86 | 202 | 1.71 | 176 | 1.64 | 199 | 1.78 | 216 | 1.77 | 270 | 1.79 | 302 | 1.82 |
| AUC(I) | 18 | 0.12 | 17 | 0.15 | 20 | 0.14 | 34 | 0.29 | 39 | 0.36 | 25 | 0.22 | 27 | 0.23 | 31 | 0.21 | 29 | 0.18 |
| AUA(M) | 189 | 1.62 | 185 | 1.60 | 134 | 1.52 | 159 | 1.51 | 166 | 1.58 | 166 | 1.58 | 156 | 1.68 | 230 | 1.67 | 200 | 1.78 |
| AUG(M) | 44 | 0.38 | 46 | 0.40 | 42 | 0.48 | 51 | 0.49 | 44 | 0.42 | 46 | 0.42 | 30 | 0.32 | 46 | 0.33 | 25 | 0.22 |
| GUU(V) | 135 | 1.86 | 140 | 1.89 | 112 | 1.77 | 121 | 1.57 | 120 | 1.71 | 159 | 2.06 | 104 | 1.86 | 111 | 1.59 | 84 | 1.46 |
| GUC(V) | 13 | 0.18 | 16 | 0.20 | 20 | 0.32 | 29 | 0.38 | 23 | 0.33 | 16 | 0.19 | 9 | 0.17 | 10 | 0.16 | 8 | 0.14 |
| GUA(V) | 112 | 1.54 | 107 | 1.43 | 87 | 1.38 | 105 | 1.36 | 88 | 1.26 | 81 | 1.05 | 88 | 1.58 | 112 | 1.62 | 118 | 2.03 |
| GUG(V) | 30 | 0.41 | 35 | 0.47 | 34 | 0.54 | 53 | 0.69 | 49 | 0.70 | 55 | 0.70 | 22 | 0.40 | 43 | 0.62 | 21 | 0.37 |
| UCU(S) | 88 | 1.98 | 114 | 2.52 | 88 | 1.84 | 72 | 1.60 | 62 | 1.42 | 73 | 1.77 | 99 | 2.39 | 104 | 2.17 | 119 | 2.31 |
| UCC(S) | 16 | 0.36 | 12 | 0.26 | 20 | 0.40 | 26 | 0.58 | 20 | 0.43 | 20 | 0.46 | 17 | 0.41 | 14 | 0.29 | 20 | 0.37 |
| UCA(S) | 60 | 1.33 | 49 | 1.10 | 49 | 1.04 | 52 | 1.17 | 52 | 1.17 | 48 | 1.16 | 56 | 1.35 | 75 | 1.59 | 62 | 1.18 |
| UCG(S) | 10 | 0.24 | 7 | 0.13 | 10 | 0.23 | 7 | 0.16 | 23 | 0.54 | 20 | 0.46 | 8 | 0.18 | 22 | 0.46 | 8 | 0.17 |
| CCU(P) | 72 | 2.09 | 75 | 2.25 | 68 | 1.93 | 68 | 1.89 | 62 | 1.88 | 68 | 1.88 | 74 | 2.27 | 64 | 1.91 | 90 | 2.52 |
| CCC(P) | 14 | 0.41 | 5 | 0.15 | 22 | 0.63 | 30 | 0.83 | 16 | 0.48 | 16 | 0.41 | 20 | 0.60 | 18 | 0.54 | 14 | 0.39 |
| CCA(P) | 46 | 1.30 | 44 | 1.30 | 42 | 1.21 | 36 | 1.03 | 44 | 1.33 | 53 | 1.46 | 34 | 1.02 | 49 | 1.49 | 34 | 0.92 |
| CCG(P) | 7 | 0.20 | 10 | 0.30 | 8 | 0.23 | 9 | 0.25 | 10 | 0.30 | 9 | 0.25 | 4 | 0.11 | 3 | 0.06 | 6 | 0.17 |
| ACU(T) | 69 | 1.76 | 100 | 2.31 | 69 | 1.70 | 74 | 1.64 | 86 | 1.74 | 72 | 1.54 | 79 | 1.97 | 72 | 1.91 | 85 | 2.24 |
| ACC(T) | 14 | 0.36 | 18 | 0.42 | 18 | 0.44 | 23 | 0.51 | 25 | 0.51 | 25 | 0.53 | 18 | 0.46 | 18 | 0.48 | 14 | 0.37 |
| ACA(T) | 68 | 1.73 | 47 | 1.09 | 62 | 1.53 | 66 | 1.46 | 66 | 1.33 | 64 | 1.37 | 53 | 1.33 | 52 | 1.40 | 45 | 1.18 |
| ACG(T) | 7 | 0.15 | 8 | 0.18 | 13 | 0.32 | 18 | 0.40 | 21 | 0.42 | 26 | 0.56 | 10 | 0.25 | 8 | 0.21 | 8 | 0.21 |
| GCU(A) | 107 | 2.38 | 122 | 2.57 | 109 | 2.04 | 72 | 1.48 | 94 | 1.93 | 62 | 1.28 | 86 | 2.11 | 85 | 2.27 | 104 | 2.38 |
| GCC(A) | 20 | 0.42 | 16 | 0.34 | 36 | 0.69 | 40 | 0.83 | 18 | 0.37 | 64 | 1.32 | 21 | 0.50 | 8 | 0.21 | 15 | 0.37 |
| GCA(A) | 36 | 0.82 | 44 | 0.93 | 56 | 1.05 | 55 | 1.15 | 56 | 1.15 | 44 | 0.91 | 51 | 1.23 | 48 | 1.28 | 39 | 0.90 |
| GCG(A) | 17 | 0.38 | 8 | 0.17 | 12 | 0.22 | 26 | 0.54 | 27 | 0.55 | 23 | 0.49 | 7 | 0.16 | 9 | 0.24 | 15 | 0.35 |
| UAU(Y) | 113 | 1.52 | 140 | 1.59 | 109 | 1.48 | 133 | 1.44 | 148 | 1.53 | 138 | 1.60 | 129 | 1.67 | 127 | 1.57 | 146 | 1.73 |
| UAC(Y) | 36 | 0.48 | 36 | 0.41 | 38 | 0.52 | 52 | 0.56 | 46 | 0.47 | 35 | 0.40 | 25 | 0.33 | 35 | 0.43 | 22 | 0.27 |
| CAU(H) | 59 | 1.71 | 49 | 1.49 | 52 | 1.53 | 49 | 1.39 | 55 | 1.33 | 55 | 1.46 | 65 | 1.65 | 66 | 1.78 | 64 | 1.73 |
| CAC(H) | 10 | 0.29 | 17 | 0.51 | 16 | 0.47 | 22 | 0.61 | 29 | 0.67 | 20 | 0.54 | 14 | 0.35 | 8 | 0.22 | 10 | 0.27 |
| CAA(Q) | 42 | 1.35 | 38 | 1.49 | 43 | 1.59 | 48 | 1.55 | 39 | 1.59 | 44 | 1.69 | 49 | 1.77 | 53 | 1.63 | 41 | 1.71 |
| CAG(Q) | 20 | 0.65 | 13 | 0.51 | 10 | 0.41 | 14 | 0.45 | 10 | 0.41 | 8 | 0.31 | 7 | 0.23 | 12 | 0.37 | 7 | 0.29 |
| AAU(N) | 103 | 1.75 | 103 | 1.72 | 90 | 1.49 | 86 | 1.31 | 88 | 1.52 | 100 | 1.65 | 112 | 1.75 | 103 | 1.63 | 129 | 1.71 |
| AAC(N) | 16 | 0.25 | 17 | 0.28 | 31 | 0.51 | 46 | 0.69 | 29 | 0.48 | 21 | 0.35 | 16 | 0.25 | 23 | 0.37 | 22 | 0.29 |
| AAA(K) | 72 | 1.71 | 60 | 1.38 | 81 | 1.78 | 55 | 1.48 | 44 | 1.21 | 66 | 1.47 | 79 | 1.60 | 81 | 1.64 | 92 | 1.84 |
| AAG(K) | 12 | 0.29 | 27 | 0.62 | 10 | 0.22 | 20 | 0.52 | 29 | 0.79 | 23 | 0.53 | 20 | 0.40 | 18 | 0.36 | 8 | 0.16 |
| GAU(D) | 52 | 1.73 | 52 | 1.70 | 51 | 1.65 | 33 | 1.10 | 42 | 1.39 | 46 | 1.48 | 40 | 1.63 | 46 | 1.48 | 48 | 1.65 |
| GAC(D) | 8 | 0.27 | 9 | 0.30 | 10 | 0.35 | 26 | 0.90 | 18 | 0.61 | 16 | 0.52 | 9 | 0.37 | 16 | 0.52 | 10 | 0.35 |
| GAA(E) | 55 | 1.33 | 49 | 1.35 | 59 | 1.51 | 47 | 1.25 | 42 | 1.24 | 49 | 1.34 | 51 | 1.53 | 48 | 1.07 | 64 | 1.52 |
| GAG(E) | 27 | 0.67 | 23 | 0.65 | 20 | 0.49 | 29 | 0.75 | 26 | 0.76 | 23 | 0.66 | 16 | 0.47 | 42 | 0.93 | 20 | 0.48 |
| UGU(C) | 35 | 1.63 | 40 | 1.67 | 29 | 1.37 | 46 | 1.64 | 42 | 1.35 | 44 | 1.80 | 36 | 1.66 | 39 | 1.70 | 35 | 1.79 |
| UGC(C) | 8 | 0.37 | 8 | 0.33 | 13 | 0.63 | 10 | 0.36 | 20 | 0.65 | 5 | 0.20 | 8 | 0.34 | 7 | 0.30 | 4 | 0.21 |
| UGA(W) | 69 | 1.38 | 65 | 1.41 | 65 | 1.57 | 49 | 1.22 | 57 | 1.31 | 56 | 1.27 | 65 | 1.52 | 77 | 1.57 | 69 | 1.51 |
| UGG(W) | 31 | 0.62 | 27 | 0.59 | 18 | 0.43 | 33 | 0.78 | 30 | 0.69 | 33 | 0.73 | 21 | 0.48 | 21 | 0.43 | 22 | 0.49 |
| CGU(R) | 16 | 1.21 | 33 | 2.17 | 9 | 0.71 | 20 | 1.29 | 23 | 1.50 | 21 | 1.33 | 17 | 1.47 | 16 | 1.18 | 15 | 1.15 |
| CGC(R) | 1 | 0.08 | 1 | 0.07 | 5 | 0.39 | 10 | 0.65 | 10 | 0.69 | 7 | 0.38 | 5 | 0.49 | 3 | 0.16 | 1 | 0.08 |
| CGA(R) | 26 | 1.96 | 16 | 1.08 | 27 | 2.12 | 17 | 1.10 | 20 | 1.19 | 18 | 1.14 | 18 | 1.63 | 27 | 2.12 | 31 | 2.38 |
| CGG(R) | 10 | 0.75 | 10 | 0.68 | 10 | 0.78 | 16 | 0.97 | 10 | 0.63 | 18 | 1.14 | 5 | 0.41 | 7 | 0.55 | 6 | 0.38 |
| AGU(S) | 65 | 1.44 | 81 | 1.75 | 66 | 1.38 | 75 | 1.71 | 74 | 1.67 | 61 | 1.48 | 49 | 1.19 | 39 | 0.81 | 34 | 0.66 |
| AGC(S) | 7 | 0.13 | 12 | 0.26 | 16 | 0.33 | 29 | 0.63 | 46 | 1.04 | 21 | 0.51 | 18 | 0.43 | 12 | 0.25 | 13 | 0.25 |
| AGA(S) | 73 | 1.62 | 59 | 1.29 | 90 | 1.88 | 53 | 1.19 | 42 | 0.95 | 59 | 1.43 | 59 | 1.42 | 74 | 1.55 | 111 | 2.13 |
| AGG(S) | 40 | 0.89 | 31 | 0.68 | 43 | 0.90 | 43 | 0.97 | 35 | 0.79 | 30 | 0.73 | 26 | 0.63 | 42 | 0.88 | 48 | 0.93 |
| GGU(G) | 91 | 1.39 | 112 | 1.98 | 70 | 1.17 | 96 | 1.54 | 109 | 1.98 | 78 | 1.21 | 77 | 1.54 | 49 | 1.00 | 46 | 0.89 |
| GGC(G) | 12 | 0.18 | 16 | 0.27 | 20 | 0.32 | 29 | 0.45 | 30 | 0.55 | 34 | 0.53 | 10 | 0.20 | 7 | 0.14 | 15 | 0.29 |
| GGA(G) | 69 | 1.06 | 48 | 0.85 | 75 | 1.26 | 55 | 0.88 | 43 | 0.78 | 72 | 1.12 | 69 | 1.38 | 83 | 1.65 | 104 | 2.00 |
| GGG(G) | 88 | 1.36 | 51 | 0.90 | 75 | 1.26 | 70 | 1.12 | 38 | 0.69 | 73 | 1.14 | 44 | 0.87 | 61 | 1.21 | 42 | 0.82 |

**Table S5.** (continued) Codon usages of the mitochondrial PCGs of stylommatophoran species.

|  | *O. dolium* | | *P. redfieldi* | | *P. bilineatus* |  | *P. cereolus* |  | *P. mexicana* |  | *P. muscorum* |  | *S. putris* | | *V. pusilla* | |
| --- | --- | --- | --- | --- | --- | --- | --- | --- | --- | --- | --- | --- | --- | --- | --- | --- |
| Codon | Count | RSCU | Count | RSCU | Count | RSCU | Count | RSCU | Count | RSCU | Count | RSCU | Count | RSCU | Count | RSCU |
| UUU(F) | 246 | 1.59 | 346 | 1.93 | 274 | 1.72 | 278 | 1.72 | 281 | 1.71 | 339 | 1.76 | 381 | 1.88 | 330 | 1.86 |
| UUC(F) | 62 | 0.41 | 12 | 0.07 | 44 | 0.28 | 46 | 0.28 | 48 | 0.29 | 46 | 0.24 | 25 | 0.12 | 25 | 0.14 |
| UUA(L) | 256 | 2.48 | 439 | 4.52 | 387 | 3.68 | 361 | 3.30 | 358 | 3.29 | 354 | 3.62 | 398 | 4.32 | 397 | 4.01 |
| UUG(L) | 83 | 0.80 | 22 | 0.23 | 55 | 0.52 | 109 | 1.00 | 111 | 1.01 | 42 | 0.42 | 44 | 0.48 | 49 | 0.49 |
| CUU(L) | 107 | 1.04 | 75 | 0.77 | 111 | 1.05 | 95 | 0.87 | 94 | 0.87 | 96 | 0.98 | 62 | 0.68 | 77 | 0.78 |
| CUC(L) | 36 | 0.35 | 1 | 0.01 | 5 | 0.05 | 16 | 0.14 | 14 | 0.13 | 10 | 0.11 | 5 | 0.05 | 18 | 0.18 |
| CUA(L) | 94 | 0.90 | 44 | 0.45 | 68 | 0.64 | 60 | 0.55 | 60 | 0.55 | 77 | 0.79 | 38 | 0.41 | 49 | 0.49 |
| CUG(L) | 44 | 0.43 | 3 | 0.02 | 7 | 0.06 | 16 | 0.15 | 17 | 0.16 | 7 | 0.07 | 5 | 0.05 | 4 | 0.04 |
| AUU(I) | 195 | 1.52 | 311 | 1.92 | 239 | 1.84 | 205 | 1.77 | 205 | 1.77 | 257 | 1.77 | 359 | 1.93 | 264 | 1.83 |
| AUC(I) | 62 | 0.48 | 13 | 0.08 | 21 | 0.16 | 26 | 0.23 | 27 | 0.23 | 34 | 0.23 | 13 | 0.07 | 25 | 0.17 |
| AUA(M) | 155 | 1.44 | 278 | 1.84 | 173 | 1.68 | 127 | 1.49 | 127 | 1.45 | 176 | 1.67 | 222 | 1.78 | 168 | 1.73 |
| AUG(M) | 60 | 0.56 | 23 | 0.16 | 33 | 0.32 | 44 | 0.51 | 48 | 0.55 | 35 | 0.33 | 29 | 0.22 | 26 | 0.27 |
| GUU(V) | 88 | 1.30 | 86 | 1.58 | 94 | 1.56 | 146 | 2.11 | 142 | 2.04 | 111 | 1.86 | 94 | 2.15 | 130 | 2.11 |
| GUC(V) | 31 | 0.46 | 9 | 0.17 | 10 | 0.18 | 14 | 0.20 | 18 | 0.26 | 20 | 0.34 | 10 | 0.25 | 13 | 0.21 |
| GUA(V) | 81 | 1.20 | 111 | 2.02 | 107 | 1.80 | 83 | 1.20 | 87 | 1.25 | 88 | 1.50 | 65 | 1.49 | 88 | 1.43 |
| GUG(V) | 72 | 1.05 | 13 | 0.24 | 27 | 0.45 | 34 | 0.49 | 33 | 0.46 | 18 | 0.30 | 5 | 0.11 | 16 | 0.26 |
| UCU(S) | 101 | 1.99 | 112 | 2.44 | 111 | 2.57 | 73 | 1.64 | 72 | 1.62 | 81 | 1.66 | 129 | 2.68 | 91 | 1.79 |
| UCC(S) | 31 | 0.60 | 8 | 0.17 | 16 | 0.35 | 30 | 0.67 | 30 | 0.68 | 16 | 0.33 | 20 | 0.39 | 14 | 0.28 |
| UCA(S) | 55 | 1.07 | 74 | 1.61 | 55 | 1.26 | 26 | 0.58 | 25 | 0.56 | 82 | 1.70 | 64 | 1.33 | 78 | 1.54 |
| UCG(S) | 20 | 0.39 | 3 | 0.04 | 8 | 0.19 | 5 | 0.11 | 5 | 0.11 | 13 | 0.27 | 10 | 0.21 | 5 | 0.10 |
| CCU(P) | 60 | 1.90 | 70 | 2.39 | 87 | 2.47 | 65 | 2.05 | 68 | 2.13 | 85 | 2.49 | 81 | 2.79 | 55 | 1.79 |
| CCC(P) | 29 | 0.89 | 3 | 0.10 | 10 | 0.28 | 16 | 0.50 | 16 | 0.47 | 12 | 0.36 | 8 | 0.28 | 8 | 0.26 |
| CCA(P) | 23 | 0.73 | 40 | 1.37 | 42 | 1.16 | 29 | 0.91 | 29 | 0.87 | 33 | 0.95 | 26 | 0.90 | 56 | 1.82 |
| CCG(P) | 16 | 0.48 | 4 | 0.14 | 3 | 0.09 | 17 | 0.54 | 17 | 0.53 | 7 | 0.21 | 1 | 0.03 | 4 | 0.13 |
| ACU(T) | 68 | 1.66 | 72 | 1.87 | 88 | 1.91 | 88 | 2.06 | 85 | 2.00 | 68 | 1.92 | 86 | 2.34 | 75 | 2.04 |
| ACC(T) | 29 | 0.72 | 10 | 0.26 | 21 | 0.45 | 33 | 0.77 | 34 | 0.80 | 18 | 0.51 | 9 | 0.24 | 9 | 0.24 |
| ACA(T) | 57 | 1.42 | 68 | 1.76 | 73 | 1.57 | 33 | 0.77 | 33 | 0.78 | 49 | 1.38 | 49 | 1.36 | 59 | 1.58 |
| ACG(T) | 8 | 0.20 | 4 | 0.11 | 3 | 0.06 | 17 | 0.40 | 18 | 0.42 | 7 | 0.20 | 3 | 0.05 | 5 | 0.13 |
| GCU(A) | 95 | 1.84 | 70 | 2.09 | 94 | 2.01 | 98 | 2.34 | 95 | 2.30 | 87 | 2.00 | 79 | 2.47 | 105 | 2.13 |
| GCC(A) | 46 | 0.89 | 8 | 0.24 | 21 | 0.45 | 31 | 0.75 | 34 | 0.82 | 23 | 0.55 | 10 | 0.34 | 25 | 0.51 |
| GCA(A) | 44 | 0.85 | 52 | 1.55 | 62 | 1.35 | 23 | 0.55 | 21 | 0.51 | 55 | 1.26 | 36 | 1.16 | 56 | 1.14 |
| GCG(A) | 22 | 0.43 | 4 | 0.12 | 9 | 0.19 | 16 | 0.36 | 16 | 0.36 | 8 | 0.18 | 1 | 0.03 | 10 | 0.22 |
| UAU(Y) | 95 | 1.16 | 178 | 1.85 | 139 | 1.73 | 152 | 1.65 | 152 | 1.66 | 107 | 1.47 | 156 | 1.76 | 107 | 1.53 |
| UAC(Y) | 69 | 0.84 | 14 | 0.15 | 22 | 0.27 | 33 | 0.35 | 31 | 0.34 | 38 | 0.53 | 21 | 0.24 | 33 | 0.47 |
| CAU(H) | 44 | 1.24 | 65 | 1.86 | 65 | 1.59 | 56 | 1.49 | 57 | 1.50 | 52 | 1.55 | 55 | 1.71 | 48 | 1.50 |
| CAC(H) | 27 | 0.76 | 5 | 0.14 | 17 | 0.41 | 20 | 0.51 | 20 | 0.50 | 16 | 0.45 | 9 | 0.29 | 16 | 0.50 |
| CAA(Q) | 36 | 1.31 | 46 | 1.76 | 59 | 1.90 | 59 | 1.73 | 56 | 1.70 | 43 | 1.76 | 44 | 1.80 | 46 | 1.61 |
| CAG(Q) | 20 | 0.69 | 7 | 0.24 | 3 | 0.10 | 9 | 0.27 | 10 | 0.30 | 7 | 0.24 | 5 | 0.20 | 10 | 0.39 |
| AAU(N) | 86 | 1.33 | 137 | 1.77 | 114 | 1.67 | 101 | 1.70 | 98 | 1.68 | 98 | 1.53 | 137 | 1.72 | 94 | 1.56 |
| AAC(N) | 43 | 0.67 | 18 | 0.23 | 23 | 0.33 | 18 | 0.30 | 20 | 0.32 | 30 | 0.47 | 22 | 0.28 | 26 | 0.44 |
| AAA(K) | 94 | 1.66 | 100 | 1.65 | 85 | 1.71 | 62 | 1.59 | 62 | 1.57 | 85 | 1.52 | 82 | 1.67 | 82 | 1.73 |
| AAG(K) | 20 | 0.34 | 21 | 0.35 | 14 | 0.29 | 16 | 0.41 | 17 | 0.43 | 27 | 0.48 | 16 | 0.33 | 13 | 0.27 |
| GAU(D) | 40 | 1.18 | 48 | 1.81 | 43 | 1.56 | 43 | 1.62 | 42 | 1.62 | 43 | 1.56 | 56 | 1.84 | 48 | 1.57 |
| GAC(D) | 29 | 0.82 | 5 | 0.19 | 12 | 0.44 | 10 | 0.38 | 10 | 0.38 | 12 | 0.44 | 5 | 0.16 | 13 | 0.43 |
| GAA(E) | 55 | 1.46 | 69 | 1.82 | 52 | 1.51 | 53 | 1.58 | 53 | 1.58 | 59 | 1.38 | 66 | 1.63 | 68 | 1.61 |
| GAG(E) | 20 | 0.54 | 7 | 0.18 | 17 | 0.49 | 14 | 0.42 | 14 | 0.42 | 26 | 0.62 | 16 | 0.37 | 16 | 0.39 |
| UGU(C) | 36 | 1.36 | 40 | 1.90 | 42 | 1.83 | 39 | 1.59 | 36 | 1.54 | 35 | 1.63 | 38 | 1.81 | 34 | 1.55 |
| UGC(C) | 17 | 0.64 | 3 | 0.10 | 4 | 0.17 | 10 | 0.41 | 10 | 0.46 | 8 | 0.37 | 4 | 0.19 | 10 | 0.45 |
| UGA(W) | 57 | 1.30 | 77 | 1.86 | 72 | 1.54 | 55 | 1.10 | 53 | 1.07 | 62 | 1.44 | 65 | 1.59 | 79 | 1.65 |
| UGG(W) | 31 | 0.70 | 7 | 0.14 | 21 | 0.46 | 44 | 0.90 | 46 | 0.93 | 23 | 0.56 | 17 | 0.41 | 17 | 0.35 |
| CGU(R) | 7 | 0.54 | 13 | 1.06 | 23 | 1.84 | 21 | 1.35 | 22 | 1.42 | 9 | 0.67 | 13 | 1.02 | 16 | 1.15 |
| CGC(R) | 8 | 0.62 | 0 | 0.00 | 4 | 0.32 | 3 | 0.19 | 3 | 0.19 | 3 | 0.22 | 0 | 0.00 | 3 | 0.15 |
| CGA(R) | 23 | 1.77 | 31 | 2.53 | 16 | 1.28 | 12 | 0.77 | 12 | 0.77 | 29 | 2.15 | 31 | 2.43 | 27 | 2.08 |
| CGG(R) | 14 | 1.08 | 5 | 0.41 | 7 | 0.56 | 26 | 1.68 | 25 | 1.61 | 13 | 0.96 | 7 | 0.55 | 8 | 0.62 |
| AGU(S) | 49 | 0.95 | 59 | 1.29 | 56 | 1.31 | 98 | 2.20 | 99 | 2.23 | 36 | 0.77 | 59 | 1.23 | 48 | 0.95 |
| AGC(S) | 36 | 0.70 | 4 | 0.09 | 12 | 0.28 | 23 | 0.54 | 23 | 0.52 | 25 | 0.52 | 12 | 0.25 | 20 | 0.37 |
| AGA(S) | 75 | 1.46 | 90 | 1.96 | 61 | 1.42 | 57 | 1.28 | 56 | 1.26 | 100 | 2.08 | 83 | 1.72 | 116 | 2.29 |
| AGG(S) | 43 | 0.84 | 18 | 0.39 | 27 | 0.63 | 44 | 0.99 | 46 | 1.01 | 33 | 0.66 | 9 | 0.19 | 35 | 0.69 |
| GGU(G) | 62 | 1.08 | 75 | 1.48 | 85 | 1.66 | 113 | 1.81 | 114 | 1.84 | 47 | 0.87 | 74 | 1.66 | 73 | 1.28 |
| GGC(G) | 36 | 0.62 | 7 | 0.14 | 7 | 0.12 | 26 | 0.42 | 27 | 0.43 | 20 | 0.35 | 7 | 0.16 | 12 | 0.21 |
| GGA(G) | 62 | 1.08 | 98 | 1.93 | 75 | 1.50 | 49 | 0.78 | 49 | 0.78 | 81 | 1.47 | 78 | 1.75 | 100 | 1.75 |
| GGG(G) | 72 | 1.22 | 23 | 0.45 | 36 | 0.73 | 62 | 0.99 | 59 | 0.94 | 72 | 1.31 | 20 | 0.43 | 43 | 0.75 |
