# Supplementary figures and images for: The complete mitogenome of Arion vulgaris Moquin-Tandon, 1855 (Gastropoda: Stylommatophora): mitochondrial genome architecture, evolution and phylogenetic considerations within Stylommatophora
Source: PeerJ. 2020 Feb 21;8:e8603. doi: 10.7717/peerj.8603 (PMC7039129; doi:10.7717/peerj.8603)

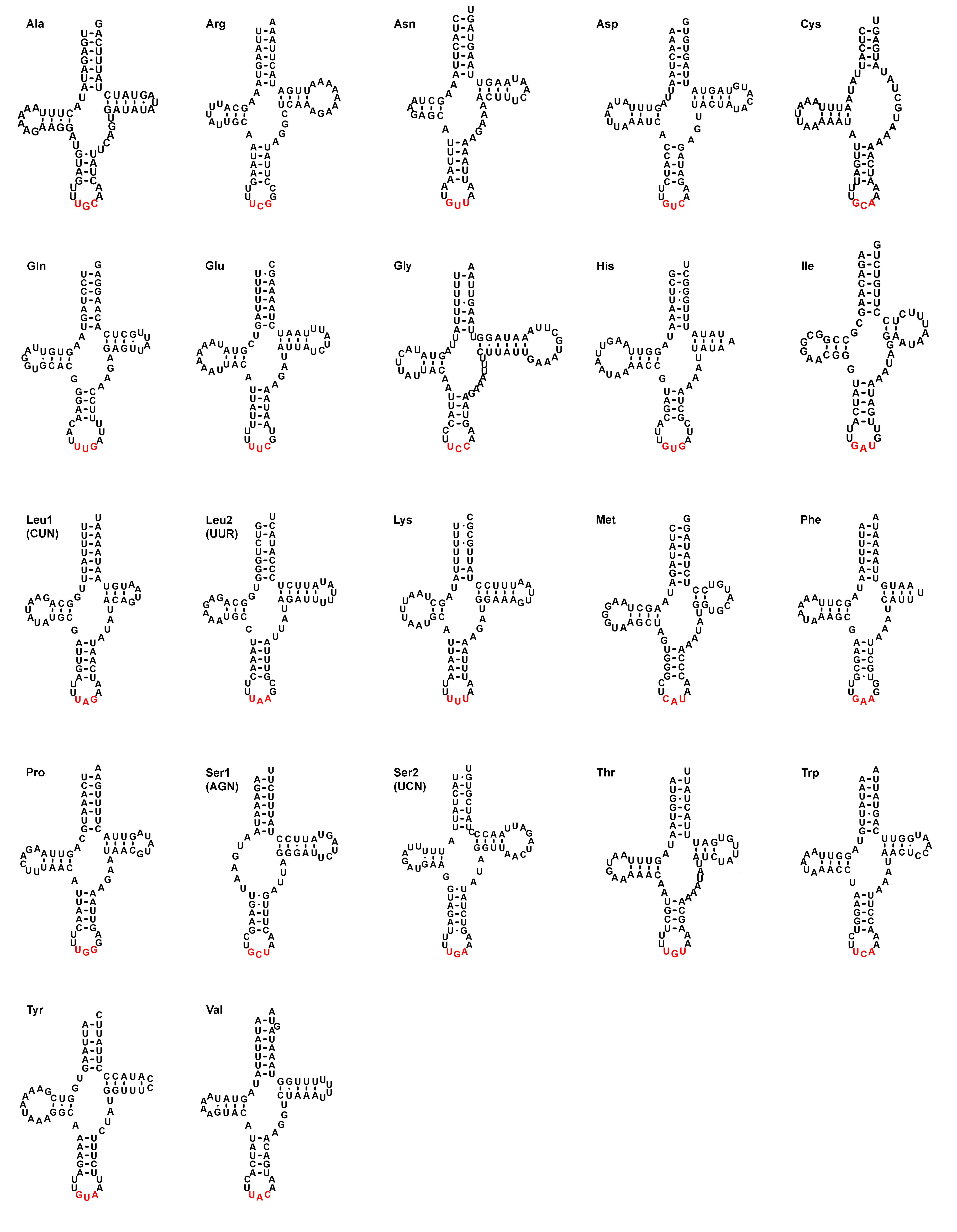

Supplement: Figure S1 [file peerj-08-8603-s006.jpg]

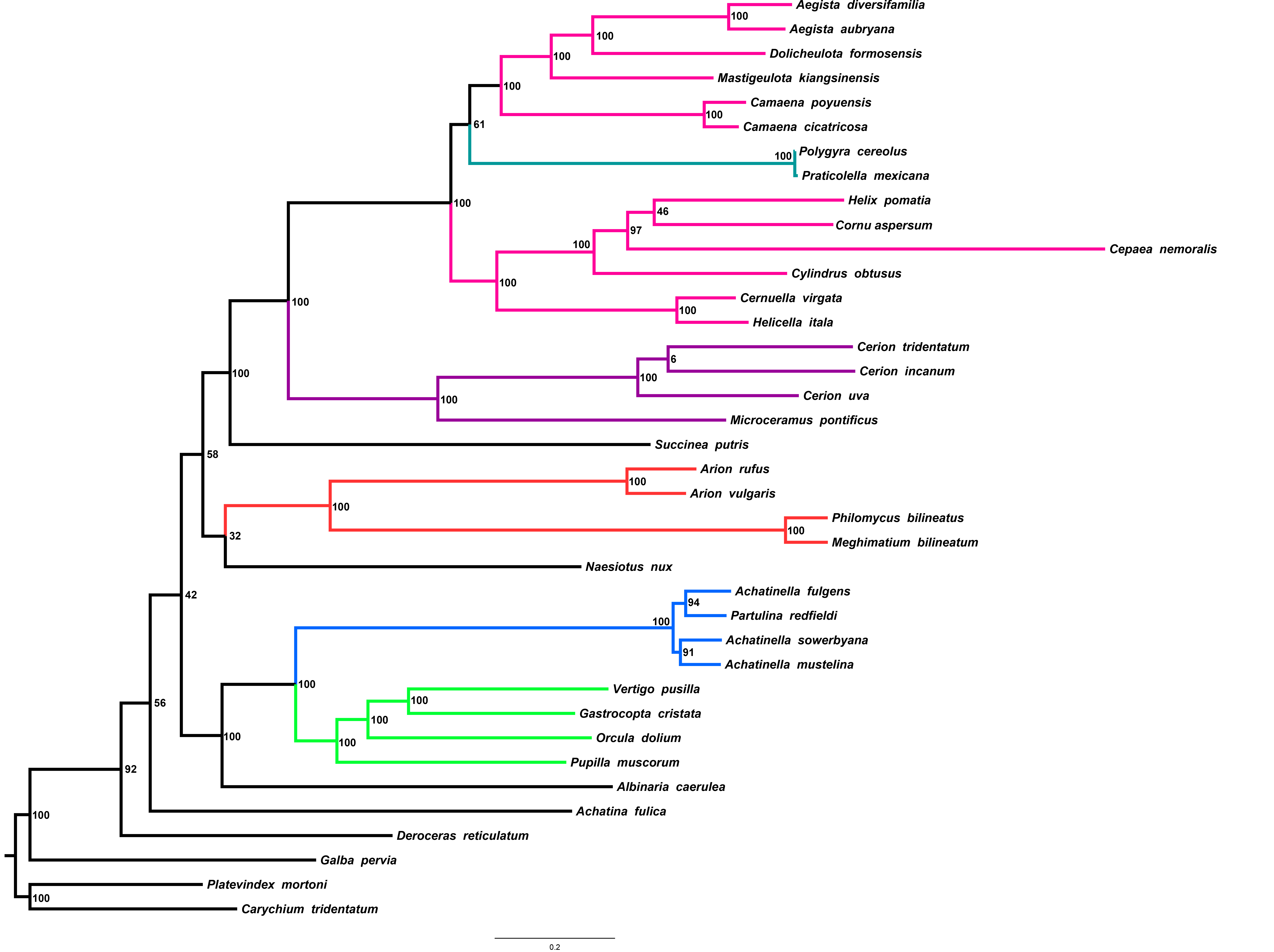

Supplement: Figure S2 [file peerj-08-8603-s007.jpg]

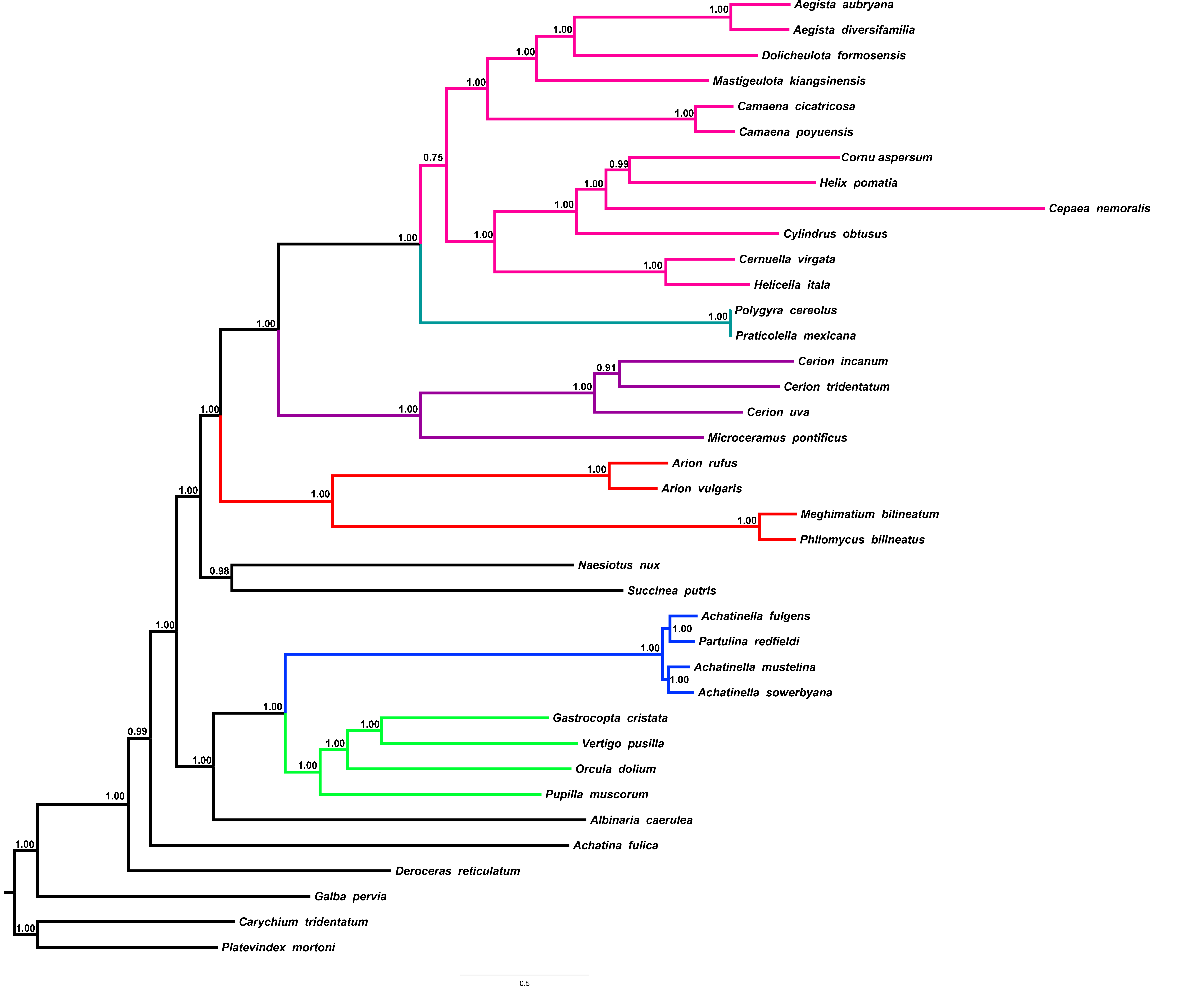

Supplement: Figure S3 [file peerj-08-8603-s008.jpg]

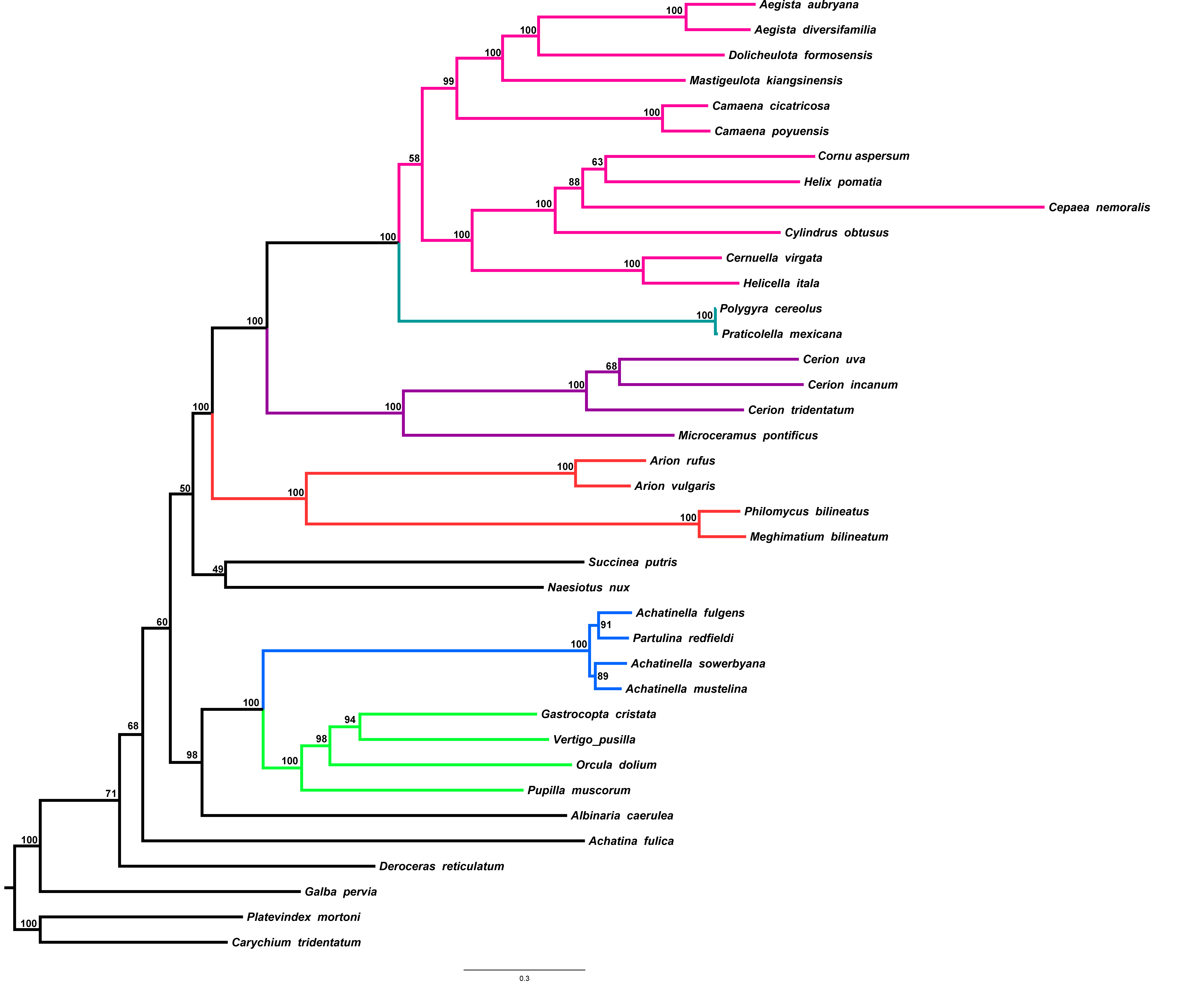

Supplement: Figure S4 [file peerj-08-8603-s009.jpg]
